# Correlation of HER2 levels expression through HER2 mRNA PCR vs immunohistochemistry (IHC) in hormone receptor positive (HR+)/HER2 negative (HER2−) early breast cancer

**DOI:** 10.1007/s12094-025-03999-7

**Published:** 2025-08-20

**Authors:** Alfonso López de Sá, Julia Tejerina, Marta Amann, Pablo Ballestín, Beatriz González, Alejandro Pascual, Cristina Díaz Del Arco, Vanesa García-Barberán, Mateo Paz-Cabezas, Alicia De Luna, José Ángel García-Sáenz, Fernando Moreno

**Affiliations:** 1 Servicio de Oncología Médica, Hospital Clínico San Carlos, Instituto de Investigación Sanitaria Hospital Clinico San Carlos (IdISSC), C/Professor Martin Lagos S/N, 28040 Madrid, Spain; 2 Clinical and Translational Research in Oncology Group, Molecular Oncology Laboratory, Hospital Clinico San Carlos, IdISSC, Madrid, Spain; 3 Servicio de Anatomía Patológica del Hospital Clínico San Carlos, IdISSC, Madrid, Spain

**Keywords:** Early breast cancer, Oncotype Dx, HER2 low, Antibody–drug conjugates

## Abstract

**Purpose:**

To evaluate the correlation between HER2 expression levels measured by HER2 mRNA using Oncotype DX and by immunohistochemistry (IHC) in hormone receptor-positive (HR+) and HER2-negative (HER2−) early breast cancer. In addition, we assessed whether low HER2 expression is associated with distinct clinicopathological characteristics and prognosis in our series.

**Methods:**

We conducted a retrospective study that included 500 patients diagnosed with stage I–III HR+/HER2− breast cancer who underwent surgery and had Oncotype DX recurrence score determined between 2009 and 2023 at Hospital Clínico San Carlos, Madrid, Spain. HER2 mRNA levels obtained through Oncotype DX were compared across IHC groups (HER2 0+, HER2 1+, HER2 2+/ISH-negative). Event-free survival (EFS) was analyzed according to HER2 expression.

**Results:**

Although HER2 mRNA levels increased with higher IHC HER2 categories, variability and overlap were observed between subgroups. Median Oncotype DX recurrence scores also rose slightly across HER2 IHC groups but did not reach statistical significance. EFS did not differ between HER2 expression levels.

**Conclusions:**

We found that HER2 mRNA measurement by Oncotype DX provides a quantitative approach to assess HER2 expression. However, its results overlap within traditional IHC categories. While HER2-low classification may have therapeutic implications for new antibody–drug conjugates, its prognostic relevance appears limited. Further studies are needed to improve HER2 quantification methods for improved clinical decision-making.

## Background

The ERBB2 oncogene encodes the human epidermal growth factor receptor 2 (HER2) protein, which plays a crucial role in breast cancer. It is associated with a poorer prognosis, yet also renders the cancer vulnerable to specific therapies.

Traditionally, HER2 expression classification and study have focused on the potential therapeutic targeting of this receptor in breast cancer. HER2 knowledge allowed the development of drugs targeting this protein. Indeed, the advent of trastuzumab, and later other anti-HER2-targeted drugs like pertuzumab or trastuzumab emtansine (T-DM1) probed the great interest of targeting HER2 in breast cancer [1–3]. However, only patients with ERBB2 amplified/HER2 overexpressed tumors experience benefit from these agents. In this regard, studies evaluating the efficacy of these drugs in tumors expressing HER2 but not meeting the mentioned criteria have been unsuccessful [4–6].

Consequently, the American Society of Clinical Oncology/College of American Pathologists (ASCO/CAP) guidelines have established criteria to identify tumors unequivocally positive for HER2 (HER2+) and, thus, suitable for anti-HER targeted therapy. These criteria are based on immunohistochemistry (IHC) methods, with a well-known scoring system ranging from 0+ to 3+, with the latter indicating intense and continuous membrane staining for anti-HER2 antibodies in over 10% of tumor cells. These tumors are considered HER2+. In addition, those with a 2+ score and positive validated in situ hybridization (ISH) techniques are also categorized as HER2+. Tumors with lower or no HER2 expression, including 0+, 1+, or 2+/ISH-negative (ISH-), are considered HER2 negative [7].

However, recent developments on the field of next-generation antibody–drug conjugates (ADC) have dramatically shifted the medical oncologist’s view regarding HER2 targeting. Indeed, the ADC trastuzumab–deruxtecan (T-DXd) has proven to be useful in metastatic HER2-negative patients whose tumors are HER2 1+ or HER2 2+/ISH−, the so-called HER2-low patients [8]. This success is possible due to the improvements in ADC crafting, such as the use of cleavable linkers that result in a bystander effect [9].

Consequently, there is an emergent need to improve the accuracy measure HER2 expression, so physicians do discard any potential candidate for receiving therapies that target HER2 expression. Traditional IHC techniques translate a continuum as discrete parameter that may potentially be outdated and insufficient to precisely define HER2 expression.

*Oncotype Dx*® is a well-stablished precision-oncology molecular assay to estimate the risk of recurrence and eventual benefit of chemotherapy for ER(+)/HER2(−) early breast cancer patients [10, 11]. Through Real-Time Quantitative Reverse Transcription polymerase chain reaction (qRT-PCR), it studies the expression of 21 cancer-related genes and provides a continuous parameter for estrogen receptor (ER) and HER2 expression [12].

Molecular analysis of breast cancers based on RNA expression could more objectively and reproducibly assess HER2 expression, further refine the prognostic or predictive value of IHC/ISH approaches, and help select patients who would benefit from treatment with HER2-targeted ADCs.

## Objectives and methods

This was a retrospective study conducted in Hospital Clínico San Carlos in Madrid, Spain. It was conducted after its approval by an ethics committee. We evaluated all consecutive patients with available *Oncotype DX* recurrence score (RS) results after surgery for stage I–III HER2-negative breast cancer from 2009 to 2023 at our institution. Patients receiving neoadjuvant chemotherapy were excluded. Tumor, patient, and treatment characteristics were obtained from medical records. Estrogen receptor (ER), progesterone receptor (PR), and HER2 status were defined according to international guidelines. HER2 status was determined using HercepTest assay.

The primary objective of the study was to correlate clinicopathological characteristics, Oncotype DX RS, and HER2 mRNA levels across the different HER2 expression groups. The secondary objective was to assess whether low-HER2 expression is associated with distinct clinicopathologic features and prognosis in patients with HR-positive/HER2-negative early breast cancer.

Qualitative variables were summarized by their number and frequencies, while age as continuous variable was summarized by the median.

Patients were stratified by their HER2 status according to the HER2 0+, HER2 1+, and HER2 2+ groups according to ASCO/CAP guidelines. Differences in age were analyzed using the Kruskal–Wallis rank sum test. Menopausal status, tumor type, grade, node involvement (*N*), and chemotherapy regimen were tested for statistical significance using Pearson’s chi-square test, while tumor size (*T*) significance was determined using Fisher’s exact test. Welch’s *F* test ANOVA was used to assess the association between the two HER2 measurement methods. Kaplan–Meier curves were constructed to evaluate event-free survival (EFS) across the different HER2 status groups, with the log-rank test applied to compare survival. False discovery rate (FDR) corrections were used to adjust for multiple testing.

All statistical analyses were performed using R (v 4.3).

## Results

### Clinical characteristics of the study population

500 patients meet the criteria to be analyzed. Among them, 169 (33.8%) had IHC 0+ tumors, 211 (42.2%) had IHC 1+ tumors, and 120 (24%) had IHC 2+/ISH-negative tumors. The median age of the overall population was 57 years (ranges 28–83). Pre- and perimenopausal patients accounted for 40% of the overall population (*n* = 192), while postmenopausal patients made up 60% (*n* = 289). The majority of tumors (81%) were of the invasive NOS subtype, with invasive lobular carcinomas comprising 13%, and other uncommon subtypes representing 6% of the cohort. Tumors ranged from 5 mm to 2 cm in size in 69% of cases, while 30% had tumors larger than 2 cm and up to 5 cm. Tumors exceeding 5 cm were uncommon (1%). Most patients (78%) had no nodal involvement, while the rest 22% had 1–3 metastatic axillary nodes.

Regarding chemotherapy, only 23% of patients received treatment, largely non-anthracycline-based regimens (19% vs. 4% of the total population), given that most patients had a low-risk recurrence score.

Clinical and pathological characteristics are detailed in Table 1.

**Table 1:** Clinical characteristics of the patients included in the series

	Category	HER2 0+	HER2 1+	HER2 2+ (FISH−)	Overall	*p* value	*q* value
Age	Age (median)	55	57	58	57	0.041	0.4
Menopausal status	Premenopausal	67 (41%)	64 (32%)	32 (27%)	163 (34%)	0.2	0.7
	Perimenopausal	10 (6%)	12 (6%)	7 (6%)	29 (6%)		
	Postmenopausal	87 (53%)	124 (62%)	78 (67%)	289 (60%)		
Type of tumor	NOS	133 (79%)	175 (83%)	96 (80%)	404 (81%)	0.8	> 0.9
	Lobular carcinoma	23 (14%)	25 (12%)	17 (14%)	65 (13%)		
	Other	13 (8%)	11 (5%)	7 (6%)	31 (6%)		
Grade	Grade 1	34 (15%)	50 (24%)	16 (13%)	100 (20%)	0.2	0.7
	Grade 2	116 (70%)	146 (70%)	94 (78%)	356 (72%)		
	Grade 3	15 (9%)	13 (6%)	10 (8%)	38 (8%)		
Tumor size (*T*)	1	113 (67%)	153 (73%)	81 (68%)	347 (69%)	0.6	0.8
	2	54 (32%)	56 (27%)	39 (32%)	149 (30%)		
	3	2 (1%)	2 (1%)	0 (0%)	4 (1%)		
Node involvement (*N*):	0	133 (79%)	159 (75%)	96 (80%)	388 (78%)	0.4	0.8
	1	35 (21%)	52 (25%)	23 (19%)	110 (22%)		
Chemotherapy	Non chemotherapy	130 (76%)	158 (75%)	95 (70%)	383 (77%)	0.9	> 0.9
	Anthracycline based	6 (3%)	9 (4%)	4 (3%)	19 (4%)		
	Non anthracycline based	33 (19%)	44 (21%)	21 (16%)	98 (19%)		

All patients had undergone surgery, and most of them had received endocrine therapy (487, 97%; *p* = 0.052; *q* = 0.4) and radiotherapy (375, 77%; *p* = 0.4, *q* = 0.8)

We found no statistically significant differences in clinicopathologic features across HER2 IHC categories in our HER2-negative population.

There was a non-statistically significant trend towards higher percentage of premenopausal patients among HER2 0+ patients. Similarly, there were no differences across subgroups concerning tumor histology, histologic grade, size, or nodal involvement.

### HER2 mRNA and RS according to HER2 IHC

**Fig. 1:**
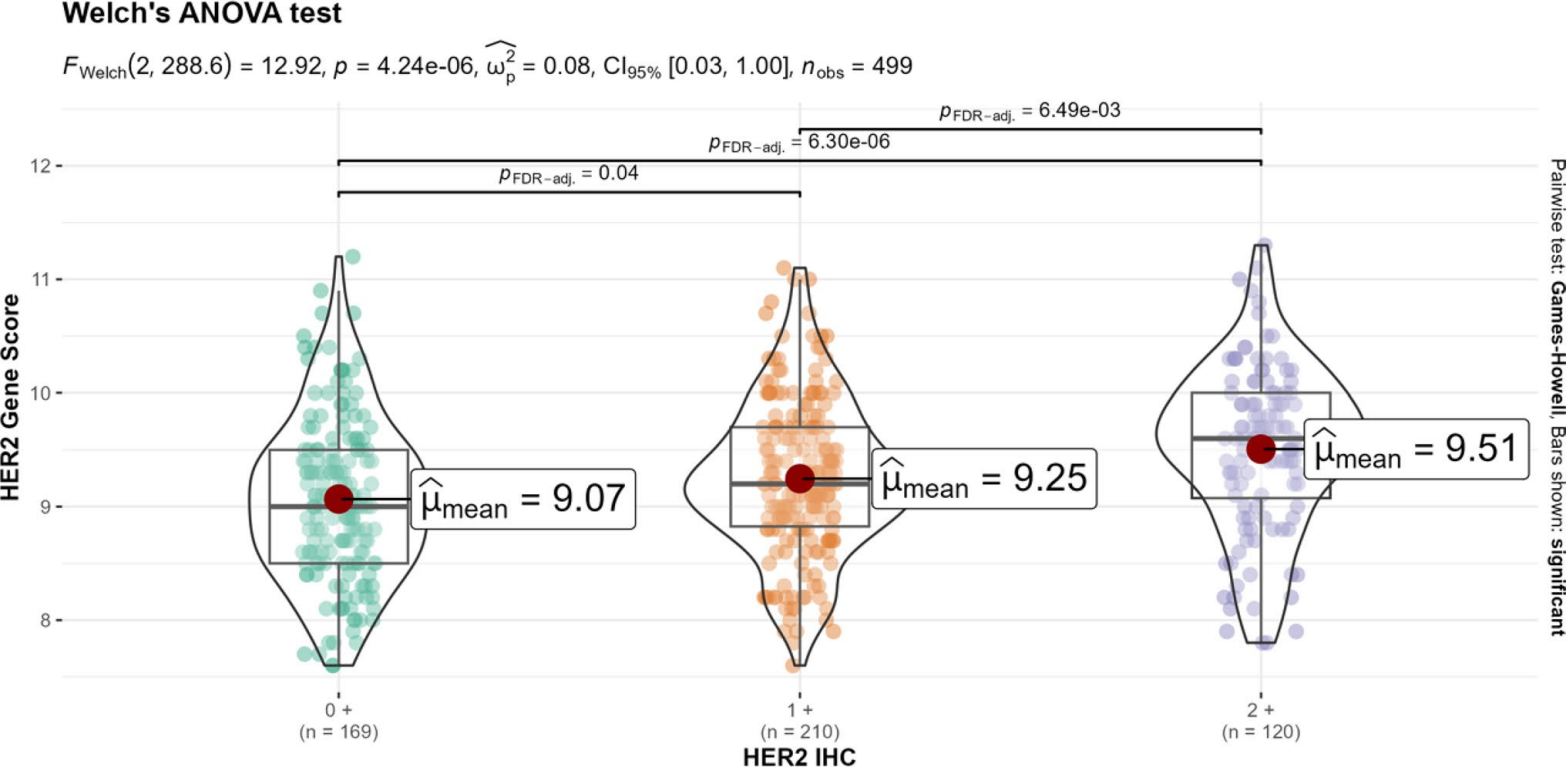
Median HER2 mRNA level according to OncotypeDx®

HER2 mRNA levels, as determined by Oncotype DX, were stratified according to IHC categories (see Fig. 1). The mean HER2 gene score increased in line with increasing IHC HER2 category. The lowest mean HER2 gene score (9.07) was observed in the HER2 0+ group, while HER2 1+ and HER2 2+/ISH-negative groups had mean scores of 9.25 and 9.51, respectively. Despite achieving statistical significance, the wide dispersion of mRNA expression across all IHC groups, and the relatively small differences in mean values, should be considered.

**Fig. 2:**
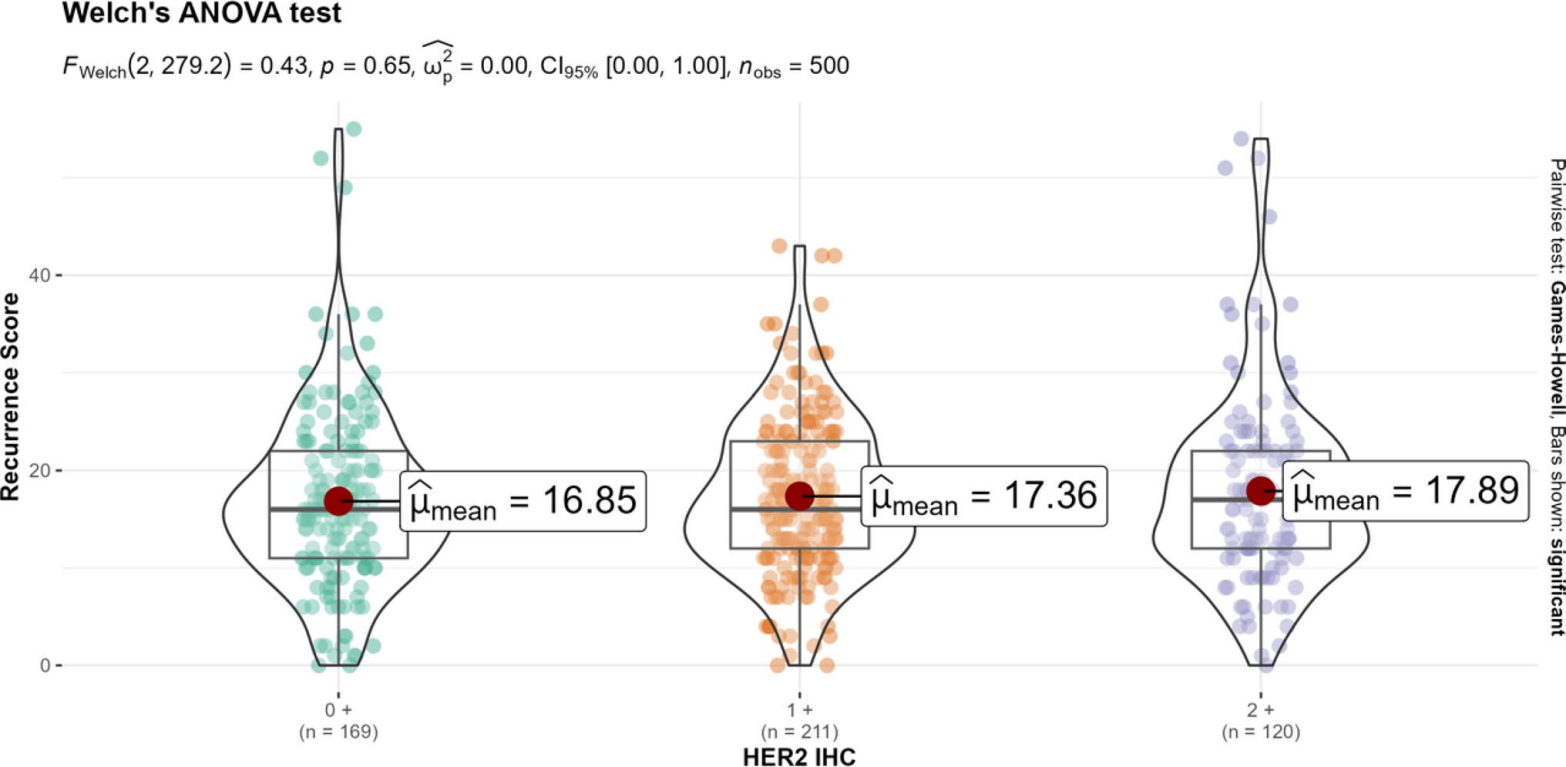
Mean OncotypeDx® recurrence score (RS) expression across the different levels of HER2 IHC expression

Mean Oncotype DX Recurrence Scores (RS) were also analyzed according to HER2 IHC categories (Fig. 2). RS increased with higher HER2 IHC categories, with medians of 16.85, 17.36, and 17.89, respectively. However, these differences did not reach statistical significance, and RS ranges were notably broad.

### Efficacy outcomes according to HER2 expression

Event-free survival (EFS) did not differ according to HER2 IHC status. With a median follow-up of 47 months, approximately 95% of patients in all cohorts remained free of any invasive event (Table 2).

**Table 2:** Presence of local or distant relapse according to HER2 IHC

Relapse	IHC 0 (*N* = 169)	IHC 1+ (*N* = 210)	IHC 2+/ISH− (*N* = 120)	Total (*N* = 499) Lost of follow up: 1 patient
No	161 (95%)	194 (92%)	116 (97%)	471 (94%)
Yes	8 (4.7%)	16 (7.6%)	4 (3.3%)	28 (5.6%)

**Fig. 3:**
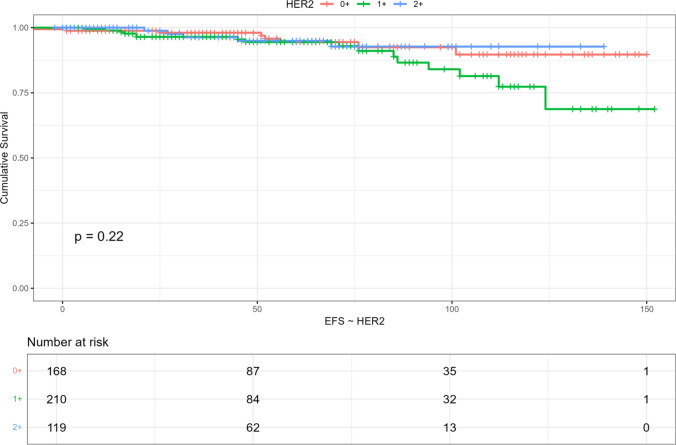
Event-free survival in the study population according to HER2 IHC

Thus far, with the above-mentioned median follow-up of 47 months, median event-free survival has not been reached, neither in the general population nor in any cohort, with no statistical significance among subtypes, as shown in Fig. 3.

## Discussion

The success of ADC-based treatments in breast cancer has underscored the importance of identifying appropriate targets to direct these drugs, maximizing their therapeutic potential [13].

Historically, beyond ER, HER2 protein overexpression has been the main therapeutic target in breast cancer due to its oncogenic role and the antitumor effects of its inhibition [14]. Consequently, HER2 ICH assays were developed with the scope of identifying tumors with HER2 overexpression.

However, this paradigm in which HER2-directed therapies require HER2 overexpression does not fully apply to current ADCs, where the membrane receptor often serves primarily as a gateway for delivering the cytotoxic payload. For detecting HER2 expression, validated immunohistochemistry (IHC) provides a semi-quantitative profile but may not fully capture the continuous variability in expression. A key limitation of IHC is its lack of reproducibility and scoring inconsistencies, particularly in 0 and 1+ categories.

Hence, the improvement of IHC techniques using HER2 mRNA expression has experienced a growing interest in the last years.

In our series, we found a statistically significant relationship between HER2 expression measured by qRT-PCR and HER2 IHC staining intensity or category, as per ASCO/CAP 2018 guidelines. Thus, transcriptomic techniques offer a quantitative and reproducible way to assess HER2 levels. Consequently, OncotypeDx showed itself capable of differentiating HER2 expression among the three HER2-negative subtypes: HER2 0+, HER2 1+ and HER2 2+/ISH negative. Our findings are coherent with previous works [15–18].

However, there was considerable variability in HER2 mRNA levels within the same IHC category, and frequent overlap between the 0+ and 1+/2+(ISH-negative) categories, limiting their clinical utility.

There are data suggesting that while T-DXd efficacy increases with higher HER2 expression, even low HER2 levels are sufficient for clinical benefit, broadening the relevance of HER2-low classification. Precise and reproducible methods for quantifying transmembrane proteins targeted by ADC antibodies are essential. In the DESTINY-Breast06 trial, the overall percent agreement for HER2-low between local and central results was 78%, with a majority of patients locally scored as HER2 IHC 0 being reclassified centrally as HER2-low (24%) or HER2-ultralow (40%). Moreover, IHC has a limited dynamic range, and some tumors with undetectable HER2 expression by IHC have mRNA levels comparable to HER2-low tumors, highlighting mRNA-based methods may enhance HER2 quantification [19].

Exploratory biomarker analysis from the DESTINY-Breast 04 study concluded that benefit in favor of T-DXd over treatment physician`s choice was observed regardless of HER2 expression levels. However, a non-significant trend for improved overall response rate (ORR) and longer progression-free survival (PFS) was observed in patients with higher versus lower HER2 gene expression levels, particularly in the T-DXd arm. Thus, HER2 levels may play a role in T-DXd efficacy among HER2-low patients [20].

The DAISY trial assessed T-DXd in HER2-positive, HER2-low, and HER2-negative (IHC 0) breast cancer. In HER2-positive cases, T-DXd showed high efficacy (ORR 70.6%, mPFS 11.6 months), consistent with DESTINY-Breast02/03 results. For HER2-low patients, significant antitumor activity was observed (ORR 37.5%, mPFS 6.7 months), aligning with DESTINY-Breast04 findings. Remarkably, in HER2-negative patients (IHC 0)—a group traditionally excluded from HER2-targeted therapies—the ORR was 29.7% and the mPFS reached 4.2 months. More recently, the DESTINY-Breast06 study confirmed T-DXd’s activity in patients with low or ultra-low HER2 expression (defined as faint, incomplete HER2 staining in ≤ 10% of tumor cells) [21].

Thus, quantitative and reproducible techniques, such as measuring HER2 mRNA expression, should be explored to better define the threshold at which trastuzumab deruxtecan becomes effective [22]. However, significant overlapping among different HER2 categories warrant further research in this field.

Our study’s second aim was to evaluate the clinicopathologic characteristics and prognosis across different HER2 expression levels. Recent interest has focused on the biological and clinical implications of tumors with low HER2 expression.

Regarding biology, we found no difference regarding RS among different HER2 IHC categories. Although there was a numerically trend towards higher RS as HER2 expression increases, this was not statically significant. This may be coherent with the hypothesis that HER2 expression beyond HER2 overexpression/amplification does not constitute an independent biological entity. Indeed, previous works regarding OncotypeDx and HER2 expression have not shown significant differences in this matter. For example, a large series conducted by Roy et al. suggested no clear difference in Oncotype Dx RS between HER2-low and HER2-zero tumors [23]. Some other series, like the one by Lin et al. did found that RS results were significantly higher in IHC 2+ vs. 0+ cases [17]. Kook et al. did found HER2-low to be an independent factor for high RS, although this finding was specifically significant among invasive ductal carcinoma histology and their series was enriched with premenopausal patients [24].

Although data remain conflicting due to the heterogeneity and retrospective nature of many studies [25], including a large retrospective study including the analysis of OncotypeDx score [19] a large meta-analysis found no significant clinical or prognostic differences between HER2-low and HER2-0 tumors [26]. In this study, clinical characteristics across the three IHC categories (HER2-positive, HER2-low, and HER2-negative) were similar in terms of all the variables based on recurrence scores. These results align with the idea that HER2 expression, beyond overexpression, does not significantly alter the biological characteristics of breast cancer. Indeed, any minor differences between HER2-low and HER2-0 tumors may be confounded by ER expression [27]. However, as previously stated, some authors have found clinical differences between HER2-low and HRE2-0. For example, Feldman et al. cohort showed that tumors > 2 cm were more likely to be HER2-low [15]. Hu et al. series found that the HER2-zero/ER+ group had significantly more grade 3 tumors than the HER2-low/ER+ group [16].

In addition, gene expression comparisons using PAM50 showed no differences between HER2-low and HER2-0 tumors in triple-negative breast cancers, and in HR+ tumors, differences were only seen in genes related to luminal differentiation. The genomic landscape of HER2-low tumors is similar to HER2-0, except for ERBB2 expression [26, 27].

From a prognostic perspective, we found no differences in event-free survival across the different IHC HER2 expression levels, indicating that biological behavior is driven mainly by proliferation and luminal differentiation genes, with HER2 IHC levels playing a limited prognostic role in the absence of HER2 amplification. It remains to be seen whether the use of cyclin-dependent kinase inhibitors in the adjuvant setting will have a distinct impact depending on HER2 expression.

The main strengths of the study include the large sample size of all consecutive incident patients meeting criteria, which provides sufficient statistical power to make comparisons and draw more robust conclusions about differences between HER2 expression groups, as well as the inclusion of clinically relevant factors such as menopausal status, tumor size, histological grade, and lymph node involvement. This offers a comprehensive view of the clinicopathologic characteristics and their relationship to HER2 expression. However, this study has several limitations. Due to its retrospective nature, the information obtained depends on the accuracy and availability of medical records. Despite finding significant differences in HER2 expression levels by IHC category, the wide dispersion of mRNA levels within each category suggests high variability, which may limit the precision of the correlation between HER2 measured by IHC and RT-PCR. In addition, it is well-known that HER2 score depends on the observer and reassessment of historical cases may led to an increase in the proportion of HER2-low. Finally, the 47-month follow-up is insufficient to reach the median EFS in any group, preventing definitive conclusions about the long-term impact of low HER2 expression on prognosis.

In conclusion, although the differentiation between HER2 0 and HER2-low tumors has little prognostic relevance, quantifying different levels of HER2 expression is important for identifying patients eligible for treatment with antibody–drug conjugates targeting this therapeutic marker. HER2 determination by RT-PCR could help define HER2 expression more precisely and reproducibly. However, further studies are needed to demonstrate its clinical utility.

## Data Availability

The data sets generated during and/or analyzed during the current study are available from the corresponding author on reasonable request.
